# Phage Therapy as a Novel Alternative to Antibiotics Through Adaptive Evolution and Fitness Trade-Offs

**DOI:** 10.3390/antibiotics14101040

**Published:** 2025-10-17

**Authors:** Song Zhang, Juhee Ahn

**Affiliations:** 1 Future Food Laboratory, Innovation Center of Yangtze River Delta, Zhejiang University, Jiaxing 314100, China; ed5988449@kangwon.ac.kr; 2 Department of Food Science and Nutrition, Zhejiang University, Hangzhou 310058, China; 3 Department of Biomedical Science, Kangwon National University, Chuncheon 24341, Republic of Korea

**Keywords:** adaptive evolution, fitness trade-offs, phage therapy, antibiotic resistance, host range expansion

## Abstract

The rapid emergence of antibiotic-resistant bacteria requires solutions that extend beyond conventional antibiotics. Bacteriophages (phages) provide targeted antibacterial action but face two key limitations: (1) their narrow natural host ranges and (2) the rapid emergence of evolved bacterial resistance. This review focuses specifically on evolved resistance and highlights two complementary strategies to overcome it by using phage-adaptive evolution and manipulating bacterial fitness trade-offs. Adaptive evolution accelerates phage/bacteria coevolution under host-mediated and environmental selective pressures such as receptor variability, bacterial resistance mutations, and nutrient limitations, resulting in phages with broader host targeting within resistant populations and enhanced lytic activity. Simultaneously, bacterial resistance to phages often leads to fitness costs, including restored antibiotic susceptibility or reduced virulence. These strategies support the rational design of phage/antibiotic combinations that suppress resistance and enhance therapeutic efficacy. In this review, we clarify the distinction between intrinsic host range limitations and evolved resistance, focusing on how adaptive strategies can specifically counter the latter. We discuss the underlying mechanisms, practical applications, and significance of this approach in clinical, agricultural, and environmental areas.

## 1. Introduction

The rapid emergence and global spread of antibiotic-resistant bacteria remain among the most serious public health challenges. In 2019 alone, antibiotic-resistant bacteria were responsible for over 1 million deaths worldwide, and this number could reach 10 million annually by 2050 without effective control measures [1,2,3,4]. This growing threat emphasizes the critical need for innovative and sustainable approaches to treat bacterial infections that have become resistant to conventional antimicrobial therapies. Multidrug-resistant (MDR) pathogens such as *Klebsiella pneumoniae*, *Pseudomonas aeruginosa*, and *Acinetobacter baumannii* have emerged as major threats, showing resistance rates greater than 90% to carbapenems, the last-resort antibiotics [5,6,7]. The failure of these antibiotics to treat infections worsens the public health crisis, especially in susceptible populations, including the elderly, immunocompromised patients, and those undergoing major surgeries [8,9]. The factors underlying the spread of antibiotic resistance are complex, including the overuse and misuse of antibiotics in both human healthcare and agriculture, environmental contamination, and insufficient diagnostic practices, all of which contribute to resistance development [9]. Antibiotic resistance can develop through various mechanisms, including genetic mutations, horizontal gene transfer, and the selective pressures exerted by repeated exposure to antibiotics [1,7,10,11]. As resistance rates continue to rise, traditional antibiotics are becoming less effective, leading to longer hospital stays, more intensive care requirements, and an increased risk of mortality. This growing resistance challenge has placed a substantial burden on healthcare systems worldwide, necessitating the exploration of alternative therapeutic approaches.

In light of this crisis, phage therapy has gained renewed attention as a potential alternative to conventional antibiotics [12,13]. Phages are viruses that specifically target and kill bacteria [12,14]. Unlike broad-spectrum antibiotics, which affect both pathogenic and beneficial bacteria, phages exhibit high specificity toward their bacterial hosts [15,16]. This specificity enables phages to target harmful bacteria and preserve the natural microbiota, providing a significant advantage over conventional antibiotics, which frequently disturb the balance of gut and skin microbiomes [13]. Additionally, phages are self-replicating at sites of infection, increasing their numbers where needed and thereby enhancing therapeutic effectiveness. However, clinical implementation is impeded by two major obstacles: the limited host range of many phages and the rapid evolution of bacterial resistance to phages that are initially effective [14]. The host range issue, in which a phage is unable to infect certain bacterial strains simply because they are not natural hosts, often leads to treatment delays as patient-specific matching is required. In contrast, evolved resistance refers to the ability of an initially susceptible bacterial host to acquire mutations or defenses that prevent infection by a previously effective phage.

Although phage therapy has been explored for decades, its overall effectiveness in clinical practice remains insufficiently demonstrated. This review therefore concentrates on evolved bacterial resistance and evolutionary strategies to address it, while acknowledging host-range limitations as contextual background. Many phages are highly specific to particular bacterial strains or serotypes, which can limit their application in polymicrobial or diverse infections. Moreover, bacteria can rapidly evolve mechanisms that prevent phage infection, for example, by modifying surface receptors or through adaptive immune systems such as CRISPR-Cas [17], and phage resistance has been reported in up to 82% of in vivo studies [18]. These observations highlights the need for approaches that both limit resistance emergence and, where appropriate, expand phage infectivity; in this work, host range expansion refers specifically to directed evolution of phages to overcome newly acquired bacterial resistance rather than to altering intrinsic initial host specificity. Recent advances in adaptive phage evolution and exploitation of bacterial fitness trade-offs provide translational strategies to develop more robust, broadly effective phage therapies by experimentally harnessing natural coevolutionary dynamics and targeting bacterial vulnerabilities.

## 2. Adaptive Evolution of Phages to Overcome Bacterial Resistance Mechanisms

**Figure 1:**
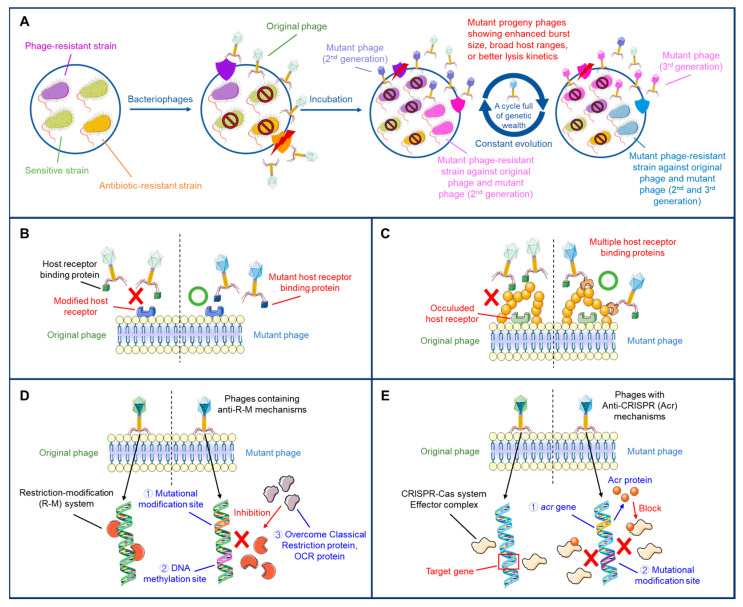
Adaptive evolution during phage/host co-cultivation and mechanisms of resistance and counter-resistance. Bacteria can counter phage invasion and evolve resistance through various mechanisms. In response, adaptive strategies are adopted by phages to overcome these defenses, resulting in dynamic co-evolution. Co-cultivation of phages and bacteria exerts selective pressure on both sides, driving adaptive evolution within microbial populations (**A**). Bacteria evade phage recognition by modifying their surface receptors, while phages adapt through mutations in their receptor-binding proteins (RBPs) to broaden recognition of these altered targets (**B**). Host cells develop surface structures that mask receptors and hinder phage attachment, while phages respond by evolving diverse receptor-binding proteins (RBPs) to ensure effective host recognition (**C**). Bacterial restriction-modification systems degrade invading phage DNA, but phages counteract these systems through genome modification, DNA methylation, or the expression of anti-restriction proteins such as OCR (**D**). Bacteria activate CRISPR-Cas systems for adaptive immunity against phages, while phages evade detection by mutating target sequences or producing CRISPR-inhibitory proteins (**E**).

Bacteria adapt to adverse environments by acquiring mutations that provide survival advantages, a process known as adaptive evolution [19,20]. This principle also applies to phages, which evolve naturally in response to bacterial resistance. Unlike genetically engineered modifications, adaptive evolution relies on the inherent biological dynamics of phages, providing a practical strategy for both basic research and field applications [21]. During phage/bacteria coevolution, bacteria develop resistance through surface receptor modifications or immune systems like CRISPR-Cas, while phages evolve new receptor specificities or anti-defense mechanisms to counter these bacterial defenses [21,22] (Table 1). Phages repeatedly exposed to diverse bacterial strains can expand their host range and overcome bacterial resistance mechanisms [21,22,23,24]. This strategy provides a promising approach for controlling antibiotic-resistant pathogens without the need for synthetic interventions (Figure 1). In addition, pre-established phage libraries provide an alternative or complementary strategy, allowing rapid selection of effective phages for specific bacteria without requiring de novo evolution, although their coverage may be limited for newly evolved resistant strains.

A novel approach to overcoming these challenges is adaptive evolution that enhances the adaptation of phages to bacterial resistance mechanisms [18,37]. Adaptive evolution, as employed in the Appelmans protocol [38], simulates bacterial resistance development under controlled conditions, enabling the rapid development of phages with expanded host ranges and enhanced lytic activity [20]. In this system, phages are introduced to bacterial populations with both sensitive and resistant strains, exposing them to selective pressures that reflect natural environments. This process leads to the evolution of phages capable of overcoming resistance mechanisms, including receptor modification, host defense systems, and changes in surface structures [22]. Through adaptive evolution, phages can evolve to infect new bacterial strains by mutating major binding proteins such as tail fibers or baseplate components. This adaptation leads to phages with expanded host ranges, enhancing their effectiveness against MDR strains [39]. Additionally, adaptive evolution can enhance the therapeutic potential of phages by increasing their burst size, improving lysis kinetics, and enabling them to target bacteria resistant to other phages [40]. However, variability in phage populations arising from natural evolution can affect reproducibility and regulatory consistency, which must be addressed in clinical applications. While some of these features have been observed in isolated studies, this review uniquely proposes a structured framework for systematically directing these adaptations to produce broadly effective and evolution-resilient therapeutic phages. Adaptive evolution can play an important role in controlling bacterial resistance by evolving phages that can recognize and bind to altered surface receptors or overcome bacterial immune defenses, providing a broader and more robust approach to phage therapy.

## 3. Adaptive Evolution and Phage/Host Interactions

Adaptive evolution lies in the fine-scale molecular interplay between phages and their bacterial hosts. The first stage of infection begins with host recognition, a process mediated by specialized receptor-binding proteins (RBPs) located on the distal ends of phage tail fibers, baseplates, or spikes [41]. These RBPs interact with specific bacterial surface structures, which can include outer membrane proteins, teichoic acids, lipopolysaccharides, capsules, or even appendages such as pili and flagella [42]. Importantly, these receptors are often essential for bacterial survival or virulence, implying that receptor modifications to evade phage infection frequently come with physiological trade-offs [22,43,44]. High-resolution structural studies, particularly those using cryo-electron microscopy, have revealed that even single amino acid substitutions in RBPs can alter host specificity, providing direct mechanistic insight into how phages can adapt to resistant hosts in short evolutionary timescales [45,46]. Once attachment occurs, the infection process proceeds through irreversible adsorption, genome delivery, and subsequent reprogramming of bacterial metabolism [47].

Phage/bacteria interactions are modulated by the dynamic physiology of bacterial cells [48]. The expression of potential receptors fluctuates with environmental conditions, nutrient availability, quorum sensing, and stress responses [48,49]. For instance, *Pseudomonas aeruginosa* downregulates porins under nutrient limitation, reducing phage entry [50], and biofilm-associated extracellular polymers shield receptors [51]. Adaptive evolution experiments that simulate these variable microenvironments such as anaerobic niches, biofilms, or host-mimicking stress conditions can generate phages capable of overcoming context-dependent barriers to infection [52]. Systems-level approaches provide powerful ways to integrate molecular insights into therapeutic design. Advances in structural biology provide atomic-level blueprints of RBPs and baseplates, which can be combined with computational protein design to guide evolution toward targeted receptor recognition [53]. Multi-omics approaches, including transcriptomics and proteomics, reveal host pathway rewiring during infection, identifying critical bacterial vulnerabilities that can be exploited in coevolution experiments [54]. Machine learning algorithms trained on large datasets of phage/host interactions are beginning to predict host specificity from genomic signatures, enabling rational pre-selection of phages most likely to evolve broad activity against resistant strains [55].

## 4. Antagonistic Pleiotropy as an Evolutionary Double-Edged Sword

**Figure 2:**
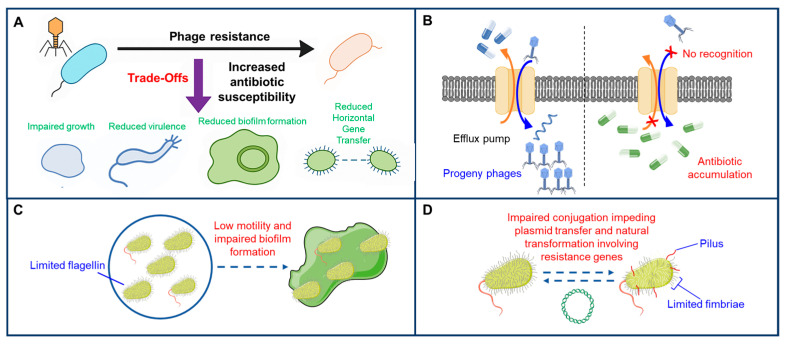
Antagonistic pleiotropy leads to fitness costs in phage-resistant bacteria, providing opportunities for targeted combination therapies. (**A**) Phage resistance in bacteria often incurs fitness costs, including impaired growth, reduced virulence, limited motility, biofilm formation defects, and reduced horizontal gene transfer. These trade-offs can increase antibiotic sensitivity and provide opportunities for targeted combination therapies. (**B**) Bacteria downregulate efflux pump-related genes to evade phage targeting. However, the loss of efflux pump function increases susceptibility to antibiotics, exposing an exploitable vulnerability. (**C**) Reduced flagellin expression helps bacteria evade phage adsorption but affects motility and disrupts biofilm formation. (**D**): Bacteria suppress fimbriae expression to prevent phage attachment, while inhibition of F-pili formation reduces conjugation and limits the spread of antibiotic resistance genes.

The evolutionary trade-offs provide opportunities to enhance the effectiveness of antibiotics when combined with phage therapy. Recent research has shown that phage-resistant bacteria often lose outer membrane proteins or receptor components, which can also play crucial roles in antibiotic efflux and general metabolism [56]. These findings indicate that phages can be deliberately selected or evolved to drive specific weaknesses in bacterial populations, thereby improving antibiotic efficacy and limiting pathogen fitness. Thus, fitness trade-offs in phage therapy design could promote synergistic effects, as phage treatment may re-sensitize bacteria to antibiotics and support more effective combination therapies [57,58]. A notable outcome of phage/bacteria coevolution is antagonistic pleiotropy, in which mutations that confer phage resistance can also increase antibiotic sensitivity, reduce bacterial virulence, or impair survival under other environmental stressors [57,59,60,61]. This phenomenon arises because adaptations that protect bacteria against phage infection often carry physiological trade-offs, limiting fitness under alternative conditions. For example, mutations in bacterial receptors that prevent phage binding may simultaneously reduce nutrient acquisition or increase susceptibility to specific antibiotics, illustrating the physiological trade-offs associated with phage resistance [42]. Specifically, phage infection can induce surface mutations affecting saccharide structures (lipopolysaccharides, teichoic acids), drug efflux transporters, or chromosomal regions that carry resistance determinants or virulence factors, generating fitness trade-offs that enhance antibiotic susceptibility. Phage resistance in bacteria often incurs fitness costs, revealing vulnerabilities that can be exploited therapeutically. These costs provide a rationale for combination strategies using both phages and antibiotics, which can push bacteria into less fit and more treatable states. Figure 2 presents key examples of these trade-offs following the development of phage resistance. In Figure 2A, resistant bacterial subpopulations often show slower growth, reduced virulence, impaired motility, or biofilm defects, while simultaneously becoming more sensitive to antibiotics, demonstrating antagonistic pleiotropy. Figure 2B shows that downregulation or loss of efflux pump-related genes impairs membrane transport and increases intracellular antibiotic accumulation [62,63,64]. In Figure 2C, reduced flagellin expression decreases motility and disrupts biofilm formation [65,66,67]. Figure 2D demonstrates that suppression of fimbriae expression limits horizontal gene transfer, restricting the spread of antibiotic resistance genes [68]. Collectively, these fitness costs represent exploitable weaknesses that can be used to enhance the efficacy of targeted combination therapies (Figure 2).

Current evidence suggests that phage therapy can restore bacterial susceptibility to certain antibiotics, particularly β-lactams and aminoglycosides, although these effects are condition-dependent and require further investigation to establish reliable clinical applications [58]. In some cases, phage resistance leads to the loss of virulence factors, producing less pathogenic bacteria [69]. These fitness trade-offs create exploitable vulnerabilities, enabling combined phage–antibiotic treatments to more effectively target resistant strains [70]. This provides a novel strategy by integrating these trade-offs into therapy design, creating intentional evolutionary bottlenecks to trap bacteria in less fit and more treatable states. By using phages to guide bacteria into these resistant but susceptible states, we can restore antibiotic efficacy and reduce the persistence of resistant strains in clinical environments (Figure 2).

## 5. Innovative Phage Therapy Through Adaptive Evolution and Fitness Trade-Offs

Adaptive evolution accelerates bacterial evolution, providing a rapid, application-driven strategy to combat antibiotic resistance by condensing long-term evolutionary changes into a defined experimental period. In vitro studies have demonstrated that phages can adapt to resistant bacterial hosts within 1 to 2 weeks under controlled laboratory conditions, providing a realistic timeframe for therapeutic development [18,71,72]. This approach advances adaptive phage solutions, enhances phage/antibiotic synergy, and predicts bacterial resistance pathways, allowing for timely and targeted interventions before resistance spreads. Such rapid adaptation has been shown to result in expanded host range and increased infectivity against previously resistant bacterial strains, thereby enabling tailored phage preparations for evolving clinical challenges. Insights into resistance dynamics across various environmental and therapeutic conditions support the design of flexible, precision-targeted phage therapies. Phage adaptation can be directed to overcome bacterial defenses, ensuring their continued effectiveness as bacterial populations evolve.

A central aspect of this strategy is taking advantage of fitness trade-offs, which occur when bacterial adaptations to one selective pressure result in reduced fitness under antibiotic exposure. This dynamic can be applied to develop therapeutic strategies that drive bacteria to lose antibiotic resistance while maintaining phage resistance. Integrating fitness trade-offs allows for the development of more effective phage therapies that target bacterial populations while also enhancing the effectiveness of existing antibiotics. The interplay between phages and antibiotics provides a complementary approach to tackling multidrug-resistant bacteria, optimizing treatment strategies, and reducing resistance development. This contributes novelty by proposing a unified evolutionary framework that simultaneously optimizes host range, lytic activity, and bacterial trade-offs to inform phage/antibiotic combinations. Primary approaches for phage/antibiotic combination design include (i) sequential administration, where phages induce resistance-associated vulnerabilities before antibiotic exposure [70], and (ii) phage cocktail approaches, combining phages targeting multiple receptors to amplify fitness costs and minimize escape mutants [61]. Sequential treatments can optimize antibiotic re-sensitization, while cocktails enhance host coverage. Additionally, antibiotic concentrations below the MIC in the presence of phages constitute an important strategy, because sub-MIC antibiotics can synergize with phages to inhibit bacterial growth and limit resistance development [73,74]. Representative examples include in vitro phage/β-lactam sequential therapy against *Pseudomonas aeruginosa* and clinical cocktail treatments for *Klebsiella pneumoniae* bloodstream infections [75,76,77,78,79]. The integration of adaptive evolution and fitness trade-offs has wide applications across preclinical, agricultural, and food settings. In clinical applications, phages developed through adaptive evolution can target multidrug-resistant pathogens, and in agriculture and food safety, phages can help reduce pathogens without promoting further resistance. This approach shows promise for treating infections caused by highly resistant pathogens such as *A*. *baumannii*, *P*. *aeruginosa*, *K*. *pneumoniae*, *Enterococcus faecium*, *Staphylococcus aureus*, *Escherichia coli*, and *Enterobacter* species, where traditional antibiotics have limited efficacy [18,37,60,67,80,81].

In agriculture, adaptive evolution-trained phages can reduce *Salmonella* contamination in poultry without relying on antibiotics, controlling antibiotic resistance in foodborne pathogens [18,37,82]. In the food industry, phages can target pathogens such as *Listeria* or *E*. *coli* without disrupting beneficial microbiota, providing a safe alternative to antimicrobials [83]. Additionally, adaptive evolution-based phages can serve as diagnostic and surveillance tools by evolving to target specific bacterial strains or resistance markers, enabling early detection of resistant pathogens in clinical or environmental samples and improving monitoring of resistance hotspots. These predictive tools could guide public health interventions and mitigate the spread of antibiotic resistance.

## 6. Challenges and Paradigm Shift in Antimicrobial Strategy

Despite its promise, the application of adaptive evolution and fitness trade-offs in phage therapy faces several challenges. Biological challenges include the unpredictability of coevolution dynamics under variable environmental conditions, the emergence of resistant subpopulations during adaptive evolution, and difficulties in maintaining phage efficacy across diverse bacterial strains. Coevolution dynamics between phages and bacteria remain complex and unpredictable, as interactions may follow diverse models based on genetic and environmental factors. Understanding these dynamics is essential for developing phages capable of overcoming resistance, ensuring sustained efficacy in the face of bacterial evolution. Furthermore, adaptive evolution for large-scale applications introduces technical challenges, including the automation of the evolutionary process and the development of high-throughput systems to rapidly generate and test phage variants. The expansion of these systems for clinical and industrial use requires the integration of real-time monitoring tools and reliable data-driven modeling to ensure reproducibility and efficiency. This approach uniquely calls for the integration of artificial intelligence, microfluidics, and evolutionary modeling to streamline and personalize phage development pipelines—an emerging interdisciplinary direction not widely discussed in prior literature. Advances in automation, microfluidics, and artificial intelligence could simplify these processes, enhancing the accessibility of adaptive evolution for efficient clinical and industrial use. Additionally, regulatory considerations regarding product consistency, safety, and characterization of evolved phages are critical, as natural adaptive evolution generates heterogeneous populations that may complicate approval processes. The development of standardized protocols for quality control, genomic stability assessment, and safety evaluation remains a major regulatory hurdle for translational implementation. Regulatory challenges, including standardizing adaptive evolution protocols for clinical phage production and ensuring the safety of evolved phages, must also be addressed before these therapies can be widely implemented. In conclusion, the integration of adaptive evolution and fitness trade-offs marks a paradigm shift in phage therapy design and application. This not only synthesizes existing findings but proposes actionable, interdisciplinary strategies that enhance the clinical readiness and translational impact of phage therapy. New paradigms, such as the intentional use of evolutionary bottlenecks to guide bacterial adaptation and AI-assisted phage selection to predict resistance pathways, represent promising future directions. While clinical applications explicitly exploiting evolutionary trade-offs are not yet established, the concept provides a framework for future therapeutic development. This shift towards evolutionary-driven phage therapy could pave the way for more sustainable and effective antimicrobial treatments in diverse settings. This approach demonstrates significant potential in clinical settings, agriculture, food safety, and environmental health. However, the successful translation of these strategies will require interdisciplinary collaboration and innovation in microbiology, synthetic biology, and bioinformatics. If these challenges are overcome, evolutionary-driven phage therapy could become a cornerstone in the global effort to reduce antibiotic resistance and sustain the effectiveness of antimicrobial treatments.

## Figures and Tables

**Table 1:** Bacterial phage resistance mechanisms and phage anti-defense strategies.

Bacterial Resistance Mechanism	Description	Phage Adaptation	Reference
Surface receptor modification	Alteration or loss of phage-binding receptors (LPS, outer membrane proteins, pili, flagella)	Mutation of tail fibers, baseplate proteins, or spikes to recognize modified or alternative receptors	[22]
CRISPR-Cas immune systems	Sequence-specific degradation of phage genomes using bacterial CRISPR arrays	Evolution of anti-CRISPR proteins or genome sequence modification to evade CRISPR targeting	[25,26]
Restriction-modification systems	Cleavage of foreign DNA at specific recognition sites	Phage DNA methylation or mutation of restriction sites to escape cleavage	[27,28,29]
Biofilm formation	Extracellular polymeric substances shielding cells and receptors	Production of depolymerases or enzymes to degrade biofilm matrix	[30,31]
Efflux pump-related resistance	Enhanced drug efflux or metabolic adaptations that indirectly limit phage entry	No direct phage adaptation; selection of phages exploiting alternative receptors or enhanced adsorption	[32,33]
Stress-response-mediated resistance	Altered receptor expression under nutrient limitation, quorum sensing, or host-mimicking stress	Selection for phages capable of infecting low-receptor-expressing or metabolically dormant cells	[34]
Receptor masking via capsules or extracellular polysaccharides	Physical shielding of receptor sites	Evolution of phages with enzymatic activity to degrade capsules or polysaccharides	[35,36]

## Data Availability

The data presented in this study are available on request from the corresponding author.
